# Why did I stop? And why did I restart? Perspectives of women lost to follow-up in option B+ HIV care in Dar es Salaam, Tanzania

**DOI:** 10.1186/s12889-019-7518-2

**Published:** 2019-08-27

**Authors:** Adellah Sariah, Joan Rugemalila, Joyce Protas, Eric Aris, Helen Siril, Edith Tarimo, David Urassa

**Affiliations:** 1 Department of Mental Health and Psychiatric Nursing, Hubert Kairuki Memorial University (HKMU), 70 Chwaku Street-Mikocheni, P.O. Box 65300, Dar es Salaam, Tanzania; 2 Department of Internal Medicine, Muhimbili National Hospital, P.O. Box 65000, Dar es Salaam, Tanzania; 3 Department of Community Health Nursing, Hubert Kairuki Memorial University (HKMU), 70 Chwaku Street-Mikocheni, P.O. Box 65300, Dar es Salaam, Tanzania; 4 Clinical Department, Management and Development for Health (MDH), P.O. Box 79810, Dar es Salaam, Tanzania; 5 Department of Public Health Evaluation, Management and Development for Health (MDH), P.O. Box 79810, Dar es Salaam, Tanzania; 6 Department of Nursing Management, Muhimbili University of Health and Allied Sciences (MUHAS), P.O. Box 65004, Dar es Salaam, Tanzania; 7 Department of Community Health, School of Public Health and Social Sciences, Muhimbili University of Health and Allied Sciences (MUHAS), P.O. Box 65015, Dar es Salaam, Tanzania

**Keywords:** Loss to follow up, Reasons, Option B +, Pregnant, Breastfeeding, HIV-positive women, Tanzania

## Abstract

**Background:**

Despite an increased uptake of option B+ treatment among HIV- positive pregnant and breastfeeding women, retaining these women in care is still a major challenge. Previous studies have identified factors associated with loss to follow-up (LTFU) in HIV care, however, the perspectives from HIV-positive pregnant and breastfeeding women regarding their LTFU in option B+ needs further exploration. We explored reasons for LTFU and motivation to resume treatment among HIV-positive women initiated in option B+ in an Urban setting.

**Methods:**

A descriptive qualitative study was conducted at three public care and treatment clinics (CTC) (Buguruni health center, Sinza hospital, and Mbagala Rangitatu health center) in Dar es Salaam, Tanzania between February and May 2017. In-depth interviews were conducted with 30 HIV-positive pregnant and breastfeeding women who were lost to follow up in the option B+ regimen. Analysis of data followed content analysis that was performed using NVivo 10 computer-assisted qualitative data analysis software.

**Results:**

Eleven women were lost to follow-up and did not resume Option B+, while 19 had resumed treatment. The study indicated a struggle with long term disease amongst HIV-positive pregnant and breastfeeding women initiated in option B+ treatment. The reported reasons contributing to LTFU among these women appeared in three categories. The contribution of LTFU in the first category namely health-related factors included medication side effects and lack of disease symptoms. The second category highlighted the contribution of psychological factors such as loss of hope, fear of medication side effects and HIV-related stigma. The third category underscored the influence of socio-economic statuses such as financial constraints, lack of partner support, family conflicts, non-disclosure of HIV-positive status, and religious beliefs. Motivators to resume treatment after LTFU included support from health care providers and family members, a desire to protect the unborn child from HIV-infection and a need to maintain a healthy status.

**Conclusion:**

The study has highlighted the reasons for LTFU and motivation to resume treatment among women initiated in Option B+. Our results provide further evidence on the need for future interventions to focus on these factors in order to improve retention in life-long treatment.

## Background

The WHO sustainable development goal (SDG) number three emphasizes healthy lives and the promotion of well-being for all ages [1]. Treatment for people living with HIV including HIV-positive pregnant and breastfeeding women fall under this SDG. The use of Option A and B treatment regimens for prevention of mother to child transmission (PMTCT) was recommended by the World Health Organization (WHO) prior to the introduction of Option B+. However, with the maintenance of viral suppression, Option B+ has proved to have important advantages over both options A and B including protection against mother to child transmission (MTCT of HIV) in subsequent pregnancies, prevention against sexual transmission to discordant couples and avoiding on and off antiretroviral treatment (ART) regimen [2].

Option B+ was introduced in Tanzania in 2013 by the Ministry of Health, Community Development, Gender, Elderly and Children (MoHCDGEC) to facilitate achievement of elimination of mother to child HIV transmission (eMTCT) goals. The treatment regimen requires that all HIV-infected pregnant and breastfeeding women are initiated on ART for life as soon as they are diagnosed, regardless of CD4 count and WHO clinical staging and 6 weeks of daily nevirapine for the infant [2]. The MoHCDGEC recommends the use of a fixed drug combination of Tenofovir/ Lamivudine/ Efavirenz (TLE) taken as one tablet a day for life. All HIV-infected pregnant women are advised to take option B+ at the beginning of the antenatal clinic and ought to continue for life.

So far, Option B+ has been rolled out in Dar es Salaam and all the other regions in the country. The life-long treatment has displayed remarkable results in resource-constrained settings; dramatically increasing the number of pregnant and breastfeeding women enrolled in ART [3]. Various reasons have been shown to elucidate why women starting ART do not attend their follow-up appointments. For instance, qualitative studies from other countries have identified factors contributing to loss to follow-up (LTFU) under the Option B+ program including lack of motivation to adhere to the life-long medication after a healthy delivery, fear of drug side effects, uncertainty on their abilities to overcome challenges of life-long medication [4, 5] and denial or lack of disclosure of HIV-positive status [6]. The former studies that implemented Option B+, [7] reported increases in ART coverage among HIV-infected pregnant women [8, 9]. However, retention in care is required for optimal prevention of negative maternal treatment outcomes and LTFU [10, 11]. Protocol changes from Option A to B and to the current B+ may have modified some of the factors associated with ART adherence or made them more pronounced. For instance, studies have reported increased problems with stigma, fear of disclosure and lack of support (emanating from the need to store and take antiretroviral (ARV) treatment more often) as well as health care system-related factors (the necessity for frequent visits to the clinic to get medication, and the limited resources and infrastructure) that pose challenges in ART implementation [6]. These problems (stigma, and fear of disclosure) continue to impede the uptake of PMTCT across sub-Saharan Africa [12].

Success in option B+ depends on the proportion of women who adhere to treatment and are retained in care, however, this is challenged by a high LTFU rate [8, 13]. Information regarding reasons for LTFU since the initiation of Option B+ in Tanzania is still scarce. Among a few available information is a report from MoHCDGEC which showed that, immediately after the introduction of Option B+, retention in care and treatment ranged from 100 to 64% on the 1st and 3rd month respectively [14]. This indicates a persistently increased rate of LTFU. We, therefore, sought to explore the reasons for LTFU among HIV-positive pregnant and breastfeeding women initiated on Option B+ in Dar es Salaam, Tanzania.

## Methods

### Design

A descriptive qualitative study was carried out between February and May 2017 to explore reasons for LTFU in Option B+ among HIV-positive pregnant and breastfeeding women.

### Setting

The study was conducted at three (1 from each of the three districts) Management and Development for Health (MDH) supported public HIV care and treatment clinics (CTC) in Dar es Salaam, Tanzania. The facilities included Buguruni health center in Ilala district; Sinza hospital in Kinondoni district; and Mbagala Rangitatu health center in Temeke district. The MDH care and treatment program was established in 2004. The program provides infrastructure, laboratory, and technical support to the five districts. Previously, the Dar es Salaam region had three districts (Temeke, Ilala, and Kinondoni). In 2016, two more districts (Ubungo and Kigamboni) were established, bringing the number of districts to five in the region.

### Sampling and recruitment of participants

Participants and the health facilities were purposively selected. The selection of health facilities was based on high patient volume. Inclusion criteria during recruitment of study participants included HIV-positive women with 18 and above years of age who were either LTFU and did not resume, or LTFU and had resumed Option B+ treatment. Participants were defined as LTFU if they had not attended the clinic for ≥180 days since the last visit [15]. Seriously ill participants and those who could not express themselves well were excluded from our study. The CTC sites determine whether patients are transferred to another clinic, either through clinic referral or self-referral to another clinic. Self-referral here means that a patient transfers oneself to another clinic rather than being referred by the health care provider. Additionally, the CTC sites usually track patients according to the guidelines that have been set by the clinic. This tracking enables health care providers to identify patients who missed appointments and attempt to re-engage them into care or identify if they had transferred to another clinic [15]. Before the recruitment of participants and data collection, two research assistants and two nurses from each of the selected clinics received a brief training that was facilitated by the principal investigator (PI). During the training, the research assistants and nurses received a brief introduction about the study objectives, purpose and ethical issues, including the informed consent process. In addition, the research assistants were trained on how to conduct in-depth interviews (IDIs), where they reviewed the data collection tool and practiced how to use it. Materials for the training included a review guide for informed consent [16], and a guide for conducting IDIs [17]. Nurses were also trained on how to identify potential participants basing on the predetermined inclusion and exclusion criteria. The nurses played an important role in identifying potential participants from the clinic files. Potential participants who met the inclusion criteria were contacted by phone. Upon identification, nurses contacted the participants, explained the purpose and benefits of the study and asked them for consent to participate in the study as well as permission to use their personal information by researchers. An appointment date for an in-depth interview (IDI) was set depending on each participant’s convenience.

### Data collection procedures

The IDIs took place at the selected clinics. One room was specifically allocated for the interviews. Participation in the study was voluntary. Before the interviews, the trained social scientists provided a full explanation to potential participants about the purpose of the study, duration and the procedures involved. This included information about voluntary participation in IDIs, audio recording procedures, the confidentiality of participants’ information and the freedom to withdraw from the study at any time without any loss of entitled services. Participants were ensured that the audio recorded interviews would not contain any of their identifying information, such as individual names. Instead, they would be identified by code numbers throughout the interviews. Participants were guaranteed that the audio recorded responses would be kept confidential and only the research team would have access to that information. Additionally, participants were ensured that any information included in the report would not identify them as respondents. Participants who agreed to participate in the study were requested to complete the written informed consent forms. IDIs were used to obtain individual perspectives on reasons for LTFU in option B+ and motivation to resume treatment after LTFU. A semi-structured topic guide in Swahili language was developed by the researcher. Before data collection, the interview guide was reviewed by PMTCT content experts to ensure that it contained all the relevant questions. It was then piloted among 5 HIV-positive women (who were not recruited for the main study) in one of the CTCs. The pilot study revealed areas that needed to be reformed. For instance, some of the questions had to be rephrased to ensure clarity during the IDIs. We revised the guide to obtain the final version for the study. Two trained social scientists (MA and KA) conducted the interviews. IDIs were audio recorded using a digital recorder and the duration for each interview ranged from 30 to 60 min. Privacy was maintained at all times during the interviews. Interviews were conducted until saturation was achieved. Saturation was reached when no new information was emerging from the interviews [18]. A maximum of two IDIs were conducted each day, data was collected for 3 weeks. A reimbursement equivalent to $2.5 was provided to each study participant as compensation for their time and travel to the clinic.

### Data analysis

Audio-recorded Swahili interviews were transcribed to text in computer files and then translated into English. The PI conducted the initial transcript examination, to obtain an overall sense of the content of responses from participants regarding various issues. Analysis of data followed qualitative content analysis [19]. Transcribed data was first served in a word processing program, i.e., Microsoft Word. Then the researcher imported the transcribed data into NVivo 10 computer-assisted qualitative data analysis software. We identified meaning units from each transcript and condensed them. We then developed a list of codes which were shared among the PI and co-authors. The team discussed, refined and agreed on a set of codes relevant for the aims of the study. The discussion provided an avenue for the addition of new codes, which were incorporated in the list of developed codes. We finally developed a common codebook to aid the analysis of data. The team used negotiation to resolve disagreements about any issue concerning the identified codes and the coding process. Each transcript was coded to gather material into nodes (categories), sub-categories and finally, the major theme emerged. An example of the content analysis process is shown in Tables 1 and 2.

**Table 1:** Example of content analysis process (Meaning unit, condensed meaning unit, and codes)

Meaning Unit	Condensed meaning unit	Codes
I stopped using medication because I had no money for bus fare and my husband left home. That is why I didn’t swallow pills for 3 months. He left home because he is not HIV-infected. The medications make me sick; I feel better without them. Actually, I do not have any symptoms, so why should I take medications?! NZo7	I stopped taking medication because my husband left me, I had no money for transport, the medications make me feel bad, and I am asymptomatic.	-Poor financial status -Lack of financial support -Lack of social support -Medication side effects -Absence of disease symptoms
I stopped taking the medication because I was not sick. How can I take drugs while I am not sick? Is that possible?... The separation from my husband also played a big part in my stopping medication. He left me because he tested HIV-negative. Therefore, I used the medication for some time, but then my legs began to feel numb …. I worry that using drugs for life may cause other problems. NZ04	I stopped the medication because I was not sick. My husband left me because he is HIV-negative. The medication caused numbness in my legs, and I feared about the problems I could get from using long-term medication	-Absence of disease symptoms -Family problems -Medication side effects -Fear of long-term effects of medications
Even though there were times did not have money for transport, I walked to the clinic to get my medication. I never thought of quitting the treatment because I wanted to protect my child. BN1	I did not have money for transport but I walked to the clinic for my medication. I wanted to protect my child	Poor financial status A need to protect the child

**Table 2:** Example of content analysis process (codes, categories, and theme)

Codes	Categories	Theme
Medication side effects	Health-related factors	Struggling with a life-long disease
Absence of disease symptoms		
Family problems	Socio-economic factors	
Poor financial status		
Lack of financial support		
Lack of social support		
Fear of long-term effects of medication	Psychological factors	
A need to protect the child	Motivation to resume treatment	

## Results

The study involved 30 HIV-positive women (12 pregnant and 18 breastfeeding). Their ages ranged between 24 and 41 years. The majority [18] had one to two children, 19 were employed, 24 had primary school level of education, and 13 were married. Eleven reported having stopped using ART (LTFU and did not resume) while 19 had resumed treatment after LTFU. Duration on ART for these women ranged from 1 to 7 years and the majority [15] had been on ART for about two to 3 years (Table 3).

**Table 3:** Characteristics of study participants

Variable	Number	Percentage
Age (years)		
20–29	7	23.3
30–39	22	73.3
≥ 40	1	3.3
Marital status		
Single	11	36.7
Married	13	43.3
Separated	3	10.0
Widowed	1	3.3
Divorced	2	6.7
Occupation		
Employed	25	83.3
Unemployed	5	16.7
Level of education		
No formal education	1	3.3
Primary school	24	80.0
Form II dropout	2	6.7
Secondary school	3	10.0
Parity		
No child	2	6.7
1–2	18	60.0
3–4	10	33.3
ART use duration (years)		
0–1	1	3.3
2–3	16	53.3
4–5	11	36.7
≥ 6	2	6.7
Duration since HIV diagnosis (years)		
0–1	1	3.3
2–3	15	50.0
4–5	10	33.3
≥ 6	4	13.3
LTFU status		
Completely LTFU	11	36.7
Resumed treatment	19	63.3

*LTFU* Loss to follow-up, *ART* Antiretroviral treatment, *HIV* Human Immunodeficiency Virus

### Themes

One main theme and four major categories emerged from this study (Table 4). Participants’ quotes are presented in the text using code numbers.

**Table 4:** Emerged categories and the main theme from the study

Categories	Theme
Health-related factors	Struggling with a life-long disease
Socio-economic factors	
Psychological factors	
Motivation to resume treatment	

Several factors were found to play a role in LTFU among HIV-positive pregnant and breastfeeding women. Participants mentioned reasons contributing to their LTFU in Option B+ as well as factors that motivated them to resume treatment. The reasons for LTFU were categorized into health-related, psychological and socio-economic factors; while motivation to resume treatment was categorized into a need to stay healthy, a need to protect the child from HIV infection and social support from family members and health care providers. There were no differences in the reasons for LTFU between breastfeeding and pregnant women. However, a major difference was observed on the motivation to resume treatment. For instance, pregnant women mainly resumed treatment because they wanted to protect their unborn children from HIV infection. On the other hand, breastfeeding women resumed treatment because they needed to maintain a healthy status, free from opportunistic infections.

### Health-related factors

Participants expressed health challenges that made them stop attending Option B+ services. Common heath concerns reported included medication side effects, larger pill sizes, and absence of disease symptoms.

### Medication side effects

Most participants could not tolerate the side effects of ART medication, particularly the first-timers. Some complained about the large pill size and how it was difficult to take them regularly as prescribed. Additionally, others worried about the outcome of their financial status because they could not continue engaging in income-generating activities due to medication side effects. A 29-year-old single woman had 2 children, was LTFU, and did not resume treatment. She had been on ART treatment under Option B+ regimen for 3 years. She reported:

*“I stopped because the medication made me feel very bad after taking them. Firstly, the tablet was too big to swallow; it would stick on my throat for hours. Secondly, it made me feel dizzy the whole day. So, I slept most of the time and could not go to work, this could have led to poverty.”* BN06

Participants expressed a variety of physical and mental disturbances when they used Option B+ medications. Common side effects including dizziness, drowsiness, confusion, and blurring of vision were reported. A 30-year-old single woman with one child was LTFU and did not resume treatment. She had been on ART under Option B+ regimen for 3 years. She described her experience as follows:

*“Whenever I took the medication, my eyes could not see properly anymore, my vision was getting weaker every day, and my head felt so heavy. I felt sick to the extent that I regretted taking the medication in the first place.”* BN05

Another participant was a 39-year-old married breastfeeding woman who had three children and had been on ART under Option B+ regimen for 4 years. She was also LTFU and did not resume treatment. She added:

*“I used to take my medication at 10 pm every night. But I would feel drunk like I had taken alcohol and I felt confused when I woke up in the middle of the night. I had to stop taking the dose.”* BN02

### Asymptomatic status

Participants claimed that they did not see the point of taking medications when they felt well, with no disease symptoms. A 30-year-old single woman with one child was LTFU and did not resume treatment. She expressed:

*“Yes, like I said I was fine until I began taking the medication. I had no signs of sickness, and I did not understand why I was supposed to take the dose while I have been fine all along.”* BN05

Other participants had been on option B+ medication ever since they were diagnosed with HIV during pregnancy. They continued taking the medication after delivery and throughout the breastfeeding period, despite the absence of disease symptoms. However, they stopped taking the medication as soon as their babies tested negative for HIV infection. A 35-year-old widow who had resumed treatment and had three children stated:

*“I tested my baby, and she was HIV-negative, then I thought I was fit and able to carry on with my daily activities, so I decided to stop taking the medication.”* NZ05

### Psychological factors

#### Loss of hope

Participants reported a loss of hope in the course of option B+ treatment, which compelled them to stop taking the medication. Some of the participants lost hope because they were abandoned by people who were expected to provide care and offer support in the important areas of their lives. For example, others were neglected by their male partners after a positive HIV diagnosis, leaving them alone with no psychological support. These women felt that they could not continue with the life-long treatment unless they received support from their significant others. Other women were burdened by the poor financial status and medication side effects which made them feel hopeless using the life-long treatment.

A 28-year-old single breastfeeding woman who had resumed treatment and had two children stated:

*“I quit taking medications because of the stress I went through. I was not feeling well …. And I was confused because the person I depended on, and who can help as well as give me courage, was not with me. I just saw myself as a dying person … ”* NZ02

A 29-year-old single woman with two children was LTFU and did not resume treatment. She said:

*“To be honest, I lost hope in the treatment because it made me feel even worse than before.”* BN06

A 33-year-old married woman who had resumed treatment and had one child added:

*“Yes, my income was so bad that I thought it is better to die even though it’s not my time yet. I lost hope back then.”* NZ08

#### Fear of medication side effects

While some participants expressed their fear of developing drug resistance as a negative outcome of long-term use of medication, others feared the immediate unpleasant and disturbing side effects of Option B+ medication. A 39-year-old married woman had three children, was LTFU and did not resume treatment expressed:

*“I fear what might happen when the treatment gets used to the body. It will fail to work and the time will come when no other medication will be useful to the body anymore.”* BN02

A single woman aging 30 years had one child, was LTFU and did not resume treatment. She reported her situation as follows:

*“… What got me worried was the way I felt sick after taking the dose. I said to myself, I was very fine before I began the treatment, but now this new treatment is going to kill me before my time. I should stop it*.” BN05

#### HIV-related stigma

Majority of participants reported that they stopped taking option B+ medication because they were scared of being identified and labeled as HIV-positive by their family members and neighbors, which could have led to stigma and discrimination. Some had to travel to the farthest CTCs away from their residencies just to avoid meeting people who would recognize them. Others decided to stop attending the CTC all together because they could not bear the thought of being identified by familiar people at the clinic. A 30-year-old married breastfeeding woman who had resumed treatment and had two children stated:

*“I developed fear and shame around people as I didn’t want anybody to know my status. Whoever enters the clinic gets the idea about your HIV status, so I decided not to go there anymore. However, after a long time, I started going to Vikindu CTC which is quite far from where I live. Nobody knows me there.”* GL05

Fear was also reported by a 34-year-old married woman who had four children. She was LTFU and did not resume treatment. She said:

*“I could not take the medication in the presence of my mother-in-law; I was afraid she would discriminate against me and tell everyone about my HIV status.”* GL10

### Socio-economic factors

#### Financial constraints

Participants had different views regarding social issues that contributed to their LTFU. Some of the participants were unemployed, and they could not afford the bus fare to attend the CTC. Others complained about the inability to buy food because of the poor economic status. With a shortage of food, they could not take their medication as required. A 32-year-old single breastfeeding woman who had one child and had resumed treatment explained her situation:

*“Yes, you may wake up early in the morning and eat nothing until 11:00 a.m. just because of economic hardship. Hence you cannot take the medication in an empty stomach.”* NZ03

Participants reported that they did not get financial support from their families or male partners which resulted in poor or non-attendance to the CTC. They also expressed their experience with the long-term treatment as they struggled to take care of themselves and their significant others amidst poor financial status. A 35-year-old married breastfeeding woman with three children was LTFU and did not resume treatment. She expressed why she dropped out of care as follows:

“*I don’t understand my husband, he knows about my HIV-positive status but refuses to give me money for transport to the clinic, he insists that I stay home.”* GL09

A 37-year-old single woman who had three children and had resumed treatment commented:

“*… … life is hard sister, there are other people I have to care for. For example, my children, even though they come from different fathers, I am grateful they not infected. But, raising them alone while still taking care of myself is a very difficult task. I am a single mother.”* GL03

#### Lack of partner support

In addition to financial problems, others felt they needed their male partner’s emotional support as they engaged in Option B+ care and treatment. However, nobody was there to comfort them during the most difficult period. Some of the participants declared that they were prohibited from attending the CTC. Therefore, lack of emotional support plus economic constraints made treatment follow-up worse. A 30-year-old woman who was separated from her husband had one child and had resumed treatment, stated:

*“I stopped using medications because my husband left me and I had no bus fare to get me to the clinic. He left me because he was HIV-negative. I, therefore, didn't take medication for three consecutive months.”* NZ07

Another 35-year-old married breastfeeding woman who had three children was LTFU and did not resume treatment, said:

*“My husband is the greatest challenge, I can’t say much, but in short, he forbids me to attend the clinic”* GL09

#### Family conflicts

Participants reported how family conflicts have contributed to their LTFU in the Option B+. For instance, conflicts between a patient and her in-laws led to anger that finally resulted in stopping the treatment. Furthermore, an HIV-negative diagnosis for some of the participants’ male partners brought conflicts in the family which led to couple separation and spouse neglect. Once the couple was separated, the women could not afford to cater for their daily needs including food and transport fee to the clinic, hence they dropped out of the Option B+ treatment. A 28-year-old married pregnant woman with no child was LTFU and did not resume treatment. She expressed:

*“I stopped because first, I traveled to my in-laws and I didn’t want my mother-in-law to know about my health status. Second, I lost my clinic card, and third, my mother-in-law wanted to break my marriage. This made me very angry; I decided to stop taking the dose completely.”* BN03

A woman of 30 years of age with one child, had resumed treatment after LTFU. She expressed how her HIV-positive status led to a separation from a husband who was HIV-negative. She stated:

*“I stopped using medications because my husband left me and I had no bus fare to get me to the clinic. He left me because he was HIV-negative. I, therefore, didn’t take medication for three consecutive months.”* NZ07

#### Lack of HIV-disclosure

Out of fear of rejection and HIV-related stigma, some of the participants explained that they stopped taking their option B+ medication because they had not told their male partners or other family members about their HIV-positive status. The lack of disclosure made it difficult to continue taking the medication or attending the clinic without being noticed or asked questions by their male partners or other family members. A 28-year-old married woman who had resumed treatment and had one child said:

*“I was afraid to disclose my status to my husband, but then, every month I had to go to the clinic for follow-up. He always asked where I was going, so it was hard for me to attend the clinic most of the time. When I finished the pills, I was given that month, I stopped and I never went to the clinic again.”* NZ01

Another woman who was 39 years of age with three children, was LTFU and did not resume treatment reported:

*“I used to stay at my mother-in-law’s place because she assisted me with the baby when I gave birth. It was very difficult for me to take the dose regularly as prescribed because I had not told her about my HIV-positive status. It reached a time when I had to quit the dose.”* BN02

#### Religious beliefs

Religious faith was mentioned as one of the reasons that made other participants stop taking their medications and attending the CTC. These women believed that only prayers could cure them of HIV infection. This was expressed by a 32-year-old single woman who had resumed treatment and had one child. She said:

*“When I was diagnosed with HIV, I started using ARVs. After birth, the baby was tested and was found HIV-negative. My husband never accepted that I was HIV-positive; he insisted that we are people of God. So, he convinced me to stop taking the ARVs, and I did. I started believing in prayers*.” NZ10

#### Motivation to resume treatment

HIV-positive women who were once LTFU felt motivated to resume treatment because they received adequate psychological support from their families and health care providers. In addition, the need to maintain a healthy status, as well as protect their children from HIV infection were among the factors that made these women decide to resume treatment.

#### The need to protect the child from HIV infection

Some of the HIV-positive women who were once lost to follow-up expressed that their decision to resume treatment was triggered by their pregnancy status. This state made them feel that they needed to protect their unborn children from HIV infection. A 36-year-old divorced pregnant woman with three children expressed:

*“Even though there were times I did not have money for transport; I walked to the clinic to get my medication. I never thought of quitting the treatment because I wanted to protect my child from HIV.”*BN1

#### The need to maintain a healthy status

Majority of women who were once LTFU resumed treatment because they wanted to keep themselves healthy, free from opportunistic infections. Some had already experienced how it feels to get opportunistic infections and they did not want to go through that again. Others had not gotten sick yet, but they wanted to remain healthy and free from infections.

A 35-year-old widowed woman with three children expressed:

*“My baby tested HIV-negative and felt that I was fit to carry out my daily activities. I decided to stop taking my medication … … As days passed, I started feeling tired most of the time and I then developed diarrhea. That’s when I decided to come to the clinic where I started taking my medication.”* NZ05

A 37-year-old single woman with three children reported:

*“I will always listen to what health care providers tell me. I will make sure that I take my medications because they keep me healthy. I will surely keep participating in the treatment, as it is for my own goodness.”* GL03

#### Social support from the family and healthcare providers

Encouragement from family members played an important role in women’ decision to start engaging in the life-long treatment after stopping. A single, 28-year-old pregnant woman with two children said:

*“I quit the medications because of the stress and what I went through. I also did not feel well, but once I told my mother, she encouraged me to go back to the clinic, where the nurse counselor gave me hope to live again.”* NZ02

Additionally, reminders and emphasis about the importance of treatment adherence and keeping ART clinic appointments by health care providers influenced women to restart treatment after LTFU. A married, 34-year-old pregnant woman with two children expressed:

“ *… . the nurses are very friendly and they keep patients’ records well. As for me, I once stopped the treatment, but I was reminded to continue and I did. I am currently expecting another child.”* GL07

## Discussion

Our study has revealed the experiences of HIV-positive pregnant and breastfeeding women as they struggle to survive while embarking in the life-long ART treatment. Common reasons for LTFU in Option B+ and factors that motivate these women to resume treatment were identified. The reasons for LTFU included health-related, psychological and social-economic factors. Motivation to resume treatment was divided into the need to maintain a healthy status, protect the child from HIV infection and support from the family and healthcare providers.

### Health-related factors

The findings demonstrated how HIV-positive women felt about ART medication. For example, their highest complaint was the issue of side effects. Similarly, previous studies have reported that women under option B+ experienced adverse medication side effects which compelled them to stop [5, 8, 14]. Apart from medication side effects, participants dropped out of the life-long treatment because they felt healthy and did not experience any disease symptoms. This is consistent with what was described in other studies [20, 21]. Additionally, similar to previous reports, some of these women dropped out of care after their children tested HIV-negative [5].

### Psychological factors

Due to the fear of anticipated HIV-related stigma, participants had to travel to the farthest clinics to receive option B+ care, which required more funds for transportation. However, a study by Iroezi et al. [22] found that women preferred clinics that were closer to their homes. The different results may have resulted from the implementation of effective interventions to reduce HIV-related stigma in those communities. Such efforts may have made HIV-positive women feel free to visit the nearest clinics for follow-up care. Consistent with other studies, loss of hope was cited as a reason for LTFU [23–25]. Interventions that focus on educating these women about the benefits of remaining in care and adhering to the treatment as well as instilling hope through counseling may help reduce the rate of LTFU in Option B+.

### Socio-economic factors

Similar to other studies, our results found that non-disclosure contributed to LTFU in option B+ treatment [12, 20, 22, 26–29]. Participants preferred to keep their HIV-positive status a secret because they feared stigma from family members, and neighbors. In the current Tanzanian society, the act of taking medication every day put the patient’s HIV-positive status at risk of being exposed, making matters worse [4]. Consistent with our findings, lack of disclosure related to HIV stigma has been reported in previous option B+ studies [22, 27, 29].

Similar to other studies, the lack of food resulting from financial constraints [22] was one of the reasons for LTFU. Participants also expressed a lack of support from their male partners [20, 26, 30]. For example, similar to previous reports [20], participants expressed how their male partners forbade their attendance to the clinics or even neglected them completely.

The role of religious beliefs in promoting behaviors that reduce new HIV infections [31] and facilitating retention in ART [32, 33] has been cited. Inconsistent with our results, religious beliefs facilitated LTFU in Option B+. Participants in our study stopped taking medications or attending the clinics because they believed they would get cured through their religious beliefs and not ART medication. Similar findings have been reported elsewhere [10, 34]. Variations in efforts taken by religious bodies to promote health-seeking behaviors among their members may have contributed to the observed inconsistencies. Perhaps, future research could explore further on how religious beliefs may influence one’s participation in long-term ART treatment. Other reasons for LTFU included traveling and relocation, which have been cited in other HIV studies [10, 30].

### Motivation to resume treatment

Studies have documented facilitators to re-engagement in ART care. For instance, consistent with our findings, most pregnant women started ART after initially stopping because they had a desire to protect their unborn babies from HIV infection [35, 36]. Another common reason for re-starting ART was feeling sick. Similar studies have cited a declined health status as a facilitator for starting ART in women under the Option B+ program [20]. In addition to feeling sick, these women re-engaged in ART care because they wanted to remain healthy [35, 37]. A healthy status enabled them to support and care for their children [36, 37]. Starting ART after LTFU was believed to improve their survival by keeping them free from opportunistic infections [36, 37]. Similar to our findings, health care providers played an important role in encouraging women who were lost to follow-up to re-engage in ART care and treatment. Such efforts acted as a motivation to resume treatment [20, 35]. Consistent with our results, these women were also motivated to resume treatment when they received support from their male partners and other members of the family [35].

### Limitations

Our data represent views of study participants from three CTCs, each located in a different district of the urban environment in Dar es Salaam. Being a qualitative study, there is a limitation in the generalizability of our findings. However, the rich information obtained from IDIs with HIV-positive pregnant and breastfeeding has provided a deeper understanding of their experiences in the life-long treatment, therefore, generating important issues that require careful consideration in PMTCT programs.

## Conclusions

Our findings provide insights on the common reasons for LTFU in women initiated on option B+ treatment. Medication side effects, feeling healthy, fear of HIV disclosure, fear of HIV-related stigma, and lack of financial and emotional support from the male partner were most commonly mentioned reasons for LTFU in option B+. Motivators for starting treatment after LTFU included support from health care providers and family members, a need to stay healthy, and a desire to protect the unborn child from HIV-infection. The study underscores the importance of strengthening HIV services by improving male partner support, promoting public awareness about negative effects of HIV-related stigma, early identification and proper management of women in ART care and treatment. Provision of psychological support for women LTFU, as well as those experiencing medication side effects, loss of hope and HIV-related fears, might help improve retention in the life-long treatment.

## Data Availability

The datasets used and analyzed during the current study are available from the corresponding author on reasonable request.

## Abbreviations

CTC: Care and Treatment Clinic
HKMU: Hubert Kairuki Memorial University
IDIs: In-depth interviews
LTFU: Loss to follow-up
MDH: Management and Development for Health
MoHCDGEC: Ministry of Health, Community Development, Gender, Elderly, and Children
MUHAS: Muhimbili University of Health and Allied Sciences
UW: University of Washington
